# Simple software solution for N_2_ diversion when measuring δ^18^O values of nitrogen-rich samples materials using a Thermo Scientific EA-IRMS System

**DOI:** 10.1016/j.mex.2023.102268

**Published:** 2023-06-22

**Authors:** Christopher Brodie

**Affiliations:** Isotrace New Zealand Limited, 167 High Street, Dunedin, 9016, New Zealand

**Keywords:** Elemental analysis, Isotope ratio mass spectrometry, Nitrogen diversion, Isobaric interference, Gas chromatography, δ^18^O value, 18O/16O ratio, Pyrolysis, m/z 30, Nitrogen-rich sample materials, N_2_ diversion when measuring δ^18^O values of nitrogen-rich samples materials

## Abstract

This method is a simple, cost-free, and reliable approach for the removal of N_2_ interference on a CO analyte when analysing nitrogen-rich (>0.5% w/w) samples by Elemental Analysis Isotope Ratio Mass Spectrometry. Specifically, the isobaric interference on *m/z* 30 is eliminated using only the open split of the Thermo Scientific ConFlo IV Universal Interface Device, improving the analytical workflow when using a static temperature Gas Chromatography (GC) column. It simplifies the N_2_ diversion methods described in recent decades. When applied, the method described here:

• Provides sufficient baseline resolution between the N_2_ and CO analytes, to permit quantitative N_2_ diversion, using an extended length packed GC column;
• Quantitatively eliminates all N_2_ analyte from the analytical gas stream ensuring that no N_2_ enters the ion source and therefore no isobaric interference is produced on *m/z* 30 ion trace of the CO analyte;
• Allows reproducible measurement of δ^18^O values from nitrogen-rich sample materials without a N_2_ isobaric interference, where the CO analyte is measured on the analytical baseline that it was produced on in the reactor (i.e., no addition make-up helium or new baseline of pure helium for the CO analyte).

## Graphical abstract

**Figure:**
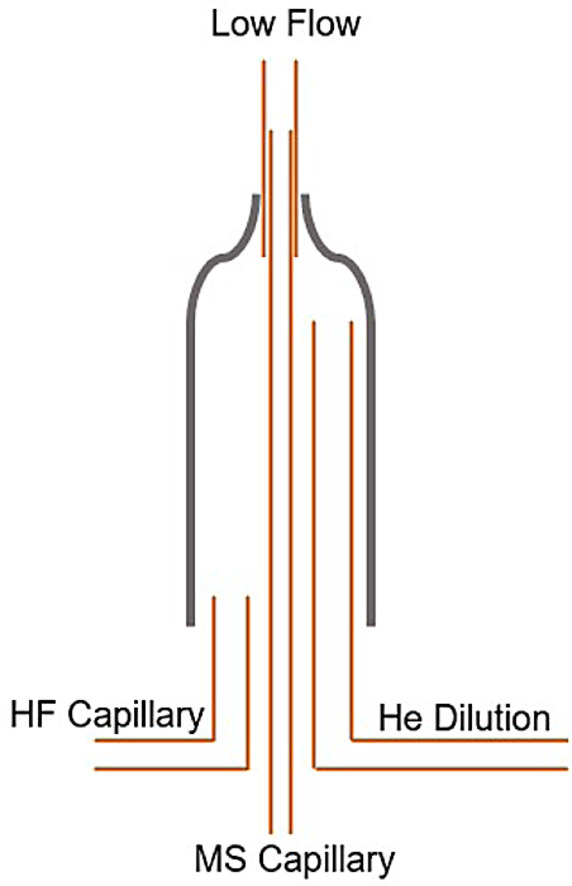

Specifications table

**Table:** 

Subject area:	Environmental Science
More specific subject area:	*Stable Isotope Analysis*
Name of your method:	*N_2_ diversion when measuring δ^18^O values of nitrogen-rich samples materials*
Name and reference of original method:	Böhlke, J.K., Mroczkowski, S.J., Coplen. T.B. Oxygen isotopes in nitrate: new reference materials for O‐18: O‐17:O‐16 measurements and observations on nitrate water equilibration. *Rapid Communications in Mass Spectrometry. 17* (2003), pp. 1835–1841.
	Brand, W.A., Coplen, T.B., Aerts‐Bijma, Q.T., Böhlke, J.K., Gehre, M., Geilmann, H., Gröning, M., Jansen, H.J., Meijer, H.A.J., Mroczkowski, S.J., Qi, H., Soergel, K.,Stuart‐Williams, H., Weise, S.M., Werner, R.A. Comprehensive inter‐laboratory calibration of reference materials for δ18O versus VSMOW using various on‐line high‐temperature conversion techniques *Rapid Communications in Mass Spectrometry.* 23 (2009), 23, pp. 999–1010.
Resource availability:	*To reproduce this method, The Analyst will require:* - *An Elemental Analyser with static temperature gas chromatography column (e.g., Thermo Scientific Flash 2000, Thermo Scientific Flash IRMS, Thermo Scientific FlashSMART)* and *an autosampler device* - *Isotope Ratio Mass Spectrometer (e.g., Thermo Scientific DELTA XP Adv; Thermo Scientific DELTA V; Thermo Scientific DELTA Q; Thermo Scientific MAT 252; Thermo Scientific MAT 253; Thermo Scientific 253 Plus)* - *Interface Device (Thermo Scientific ConFlo IV Universal interface Device)* - *To meet the manufacturer operational criteria for the above hardware* - *Isodat Software Suite 3.0* - *A computer System controlling the hardware and software listed above* - *A spreadsheet software package for data summary and evaluation* - *Nitrogen-rich sample material containing oxygen, homogenized in a manner suitable for stable isotope analysis* - *Nitrogen-rich, matrix matched quality control materials, that contain oxygen, and suitable isotope reference materials for delta-scale normalisation* - *Sample material preparation equipment, which may included but is not limited to, ball mill/grinder, oven; freeze dryer; sampling vials* - *Microbalance and weighing tools (e.g., spatula; tweezers)* - *Silver capsules and sample well tray with cover* - *Extended length Gas Chromatography column (ideally >1.2* *m length) with at least 60–80 mesh 5* *Å molecular sieve packing.*

## Method details

### A simplified solution for N_2_ diversion

The method described here is a simple, cost-free, and reliable approach for the removal of N_2_ interference on a CO analyte when analysing nitrogen-rich sample materials by Elemental Analysis Isotope Ratio Mass spectrometry (EA-IRMS) with a static temperature Gas Chromatography (GC) column. This N_2_ analyte diversion solution is achieved using software parameters only and generally does not require new hardware, hardware modifications, or any software scripting/programming knowledge. Additionally, the method described herein removes the requirement for the trial and error often needed when stabilizing baseline resolution of the N_2_ and CO analytes through optimisation of the helium carrier flow rate, GC column temperature, GC column length and GC column packing material [1]. The solution is therefore easy to implement and ensures that the sample CO analyte remains on the same, contextual background from the point of evolution in the reactor through to ion counting. The focus of this method is to describe, step-by-step, what is required to permit the method's execution: the description provides scope for refinement by The Analyst as they may need for their local laboratory protocols.

The solution is achieved using the existing capabilities of the ConFlo IV Universal Interface Device (Thermo Fisher Scientific Bremen, Germany) in conjunction with the Isodat Software Suite 3.0.94.12 (Thermo Fisher Scientific Bremen, Germany). At the time of writing, this method cannot be transferred/implemented in the Thermo Scientific Qtegra ISDS software platform due to limited control of the ConFlo IV Interface Device technical features, and cannot be implemented on the ConFlo I, II or III in Isodat 3.0 for the same reasons.

In this method, The Analyst will be required to set (i) the GC column temperature; (ii) the EA carrier gas flow rate; (iii) the timing of the sample split mass spectrometer capillary being set to low flow mode in the ConFlo IV and (iv) the timing of the high flow capillary being exposed to the mass spectrometer capillary. Setting these parameters will ensure the N_2_ analyte, produced from nitrogen-rich samples intended for the measurement of δ^18^O values of those samples, is eliminated from the gas stream. In removing the N_2_ analyte, this will eliminate a known artefact on measured δ^18^O values (see Additional Information). The following sections offer explicit guidance on how to successfully implement this workflow and assume no prior knowledge from the reader.

## PART 1: Configuring the ConFlo IV Device

**Fig. 1:**
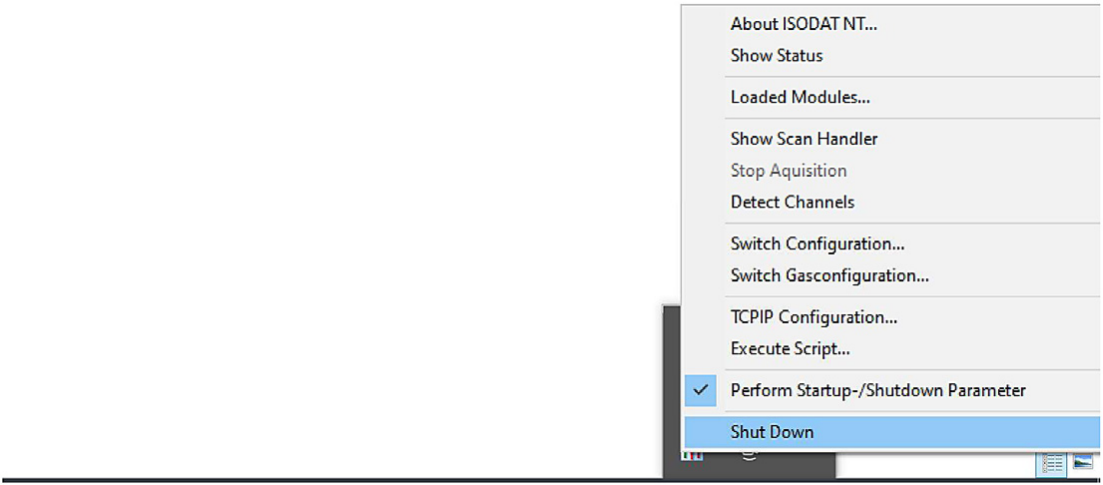
Isodat System Shut Down.
*STEP 2:* Open the Isodat Configurator and activate advanced mode. To activate advanced mode, locate ``Edit'' in the taskbar, click on it, and then click on ``Advanced mode'' from the drop-down list (Fig. 2a): a new dialogue box will appear with a message and an option to select ``OK''. The Analyst should select ``OK'' and the dialogue box will disappear and the Advanced Mode will be activated. New tabs will appear in the main configurator window (see Fig. 2b).

The ConFlo IV Device requires additional valve and capillary control options to be visible in the Isodat time events list of Method Files (*.met). Adding these event timed options to the time events list can only be achieved through the Isodat Configurator. The Analyst is required to follow these steps to configure the time events list for this method (*note:* the author assumes The Analyst already has an EA-IRMS configuration set-up in Isodat: if one is not setup, this ideally should be done first):

*STEP 1:* Close Isodat Software Suite completely. The Isodat Configurator cannot be opened whilst any of the Isodat processes are operating. Before closing Isodat, The Analyst should ensure all work/files are saved. To shutdown Isodat and prepare to open the Isodat Configurator, The Analyst must close all open Isodat windows. After closing all windows, navigate to the Windows Taskbar (bottom right-hand side of the screen), and locate the Isodat System Icon, right click on it and select ``Shut Down'' (See Fig. 1).

**Fig. 2a:**
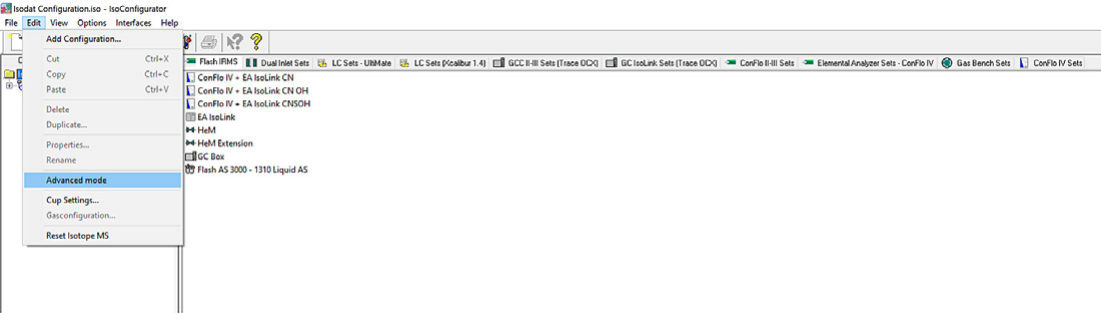
Setting Advanced Mode in Isodat Configurator.

**Fig. 2b:**
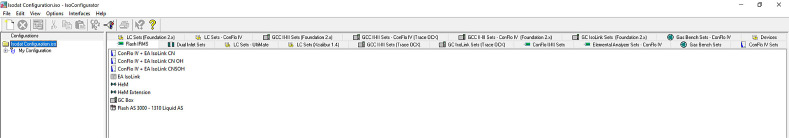
View of Advanced Mode in Isodat Configurator.
*STEP 3:* Now in Advanced Mode, navigate to the ``Devices'' tab, which is a newly appeared tab in advanced mode, and find the ``ConFlo IV Interface'' icon. Once the ``ConFlo IV interface'' icon has been located, right click on the icon and select ``Edit'' (Fig. 3a).

**Fig. 3a:**
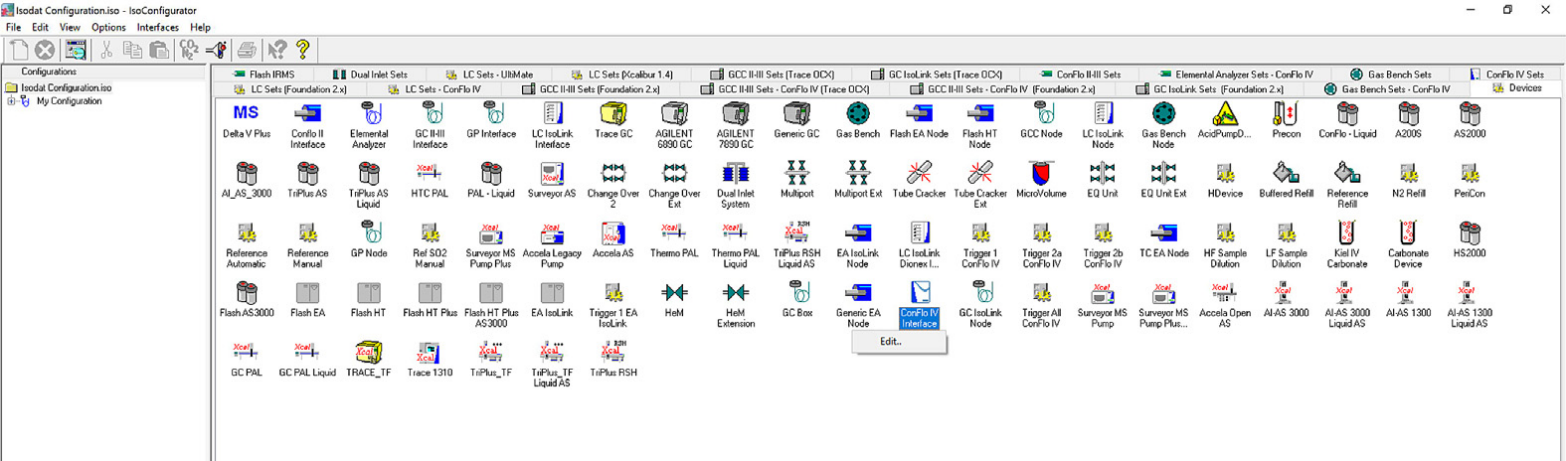
Locating ``Edit'' option for ConFlo IV Device.

Once ``Edit'' has been selected, a new dialogue box will open as shown in Fig. 3b. This dialogue box will contain several tabs that can be navigated through by The Analyst.

**Fig. 4:**
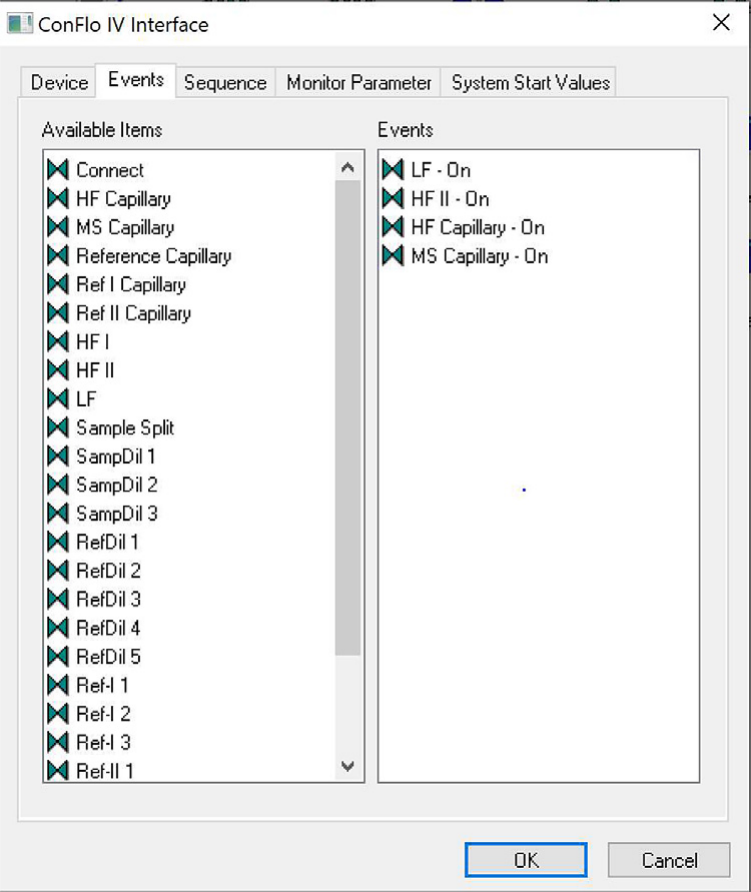
Configured Events list for ConFlo IV Interface Device.

*STEP 4:* Navigate to the Events tab. In the Events tab, there are two columns. From the left-hand list of ``Available Items'', one by one, drag and drop ``HF Capillary'', ``MS Capillary'', ``LF - On'' and either ``HF I - On'' or ``HF II - On'' to the right-hand list, labeled ``Events'' (see Fig. 4 and Analyst Tip below). The Analyst can drag and drop each required item by clicking and holding the left mouse button over the item in the ``Available items'' list, dragging it across to the ``Events'' list, and releasing the left mouse button: this will place the selected item into the ``Events'' list. Once the right-hand list has been populated with the four options stated above, click ``OK''. Clicking ``OK'' will save the additions to the Events list. The Analyst can now exit the Isodat Configurator by clicking the cross in the top right-hand corner of the Configurator window.

**Fig. 3b:**
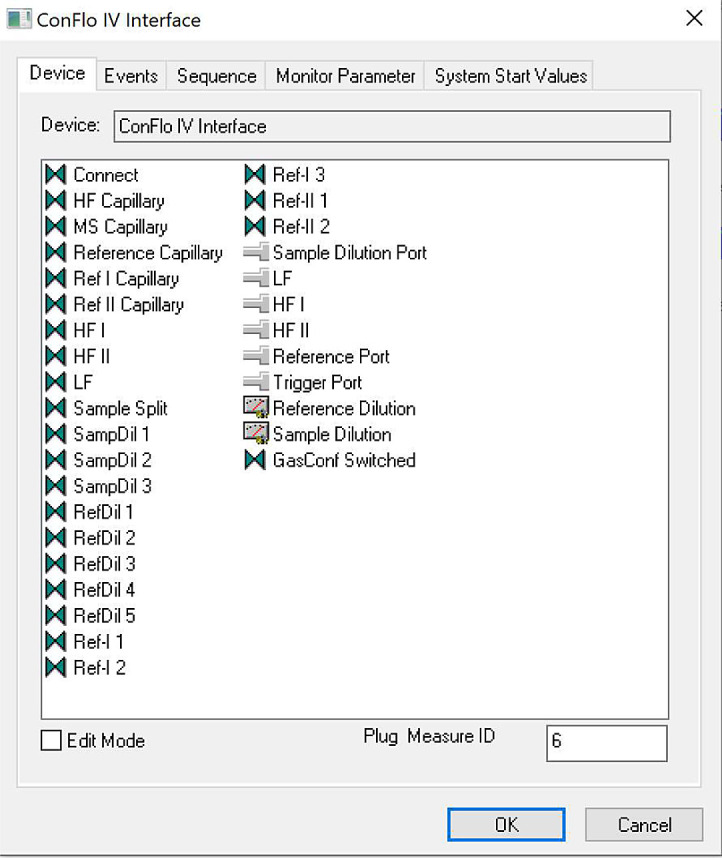
ConFlo IV Interface Advanced Settings Module.

*ANALYST TIP*: The Analyst must check which High Flow port their EA is physically connected too. This can be done by looking at the rear panel of their ConFlo IV Interface Device, and noting which port, HF I or HF II (as labeled on the ConFlo Device Rear Panel), the EA is connected too. If in doubt, The Analyst should refer to their ConFlo Interface Device Manual for further description on the rear panel and associated safety guidance and instructions.

## PART 2: N_2_ diversion method parameters

**Fig. 5:**
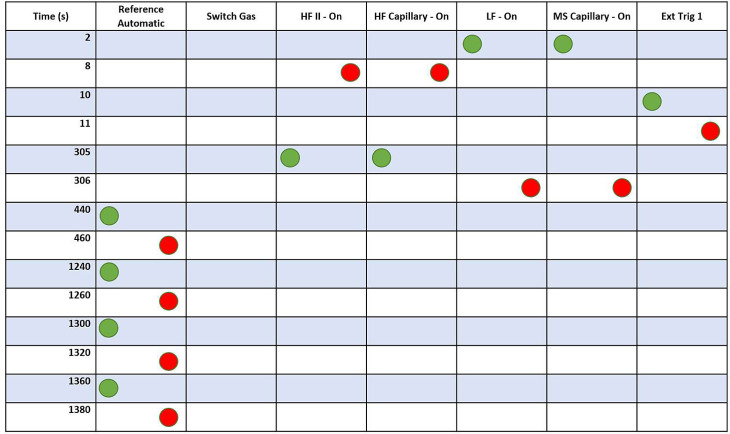
Isodat Time events list for N_2_ diversion method.

The method embodiment described herein requires the parameters detailed for the Flash/EA IsoLink OH (Table 1) or the TC/EA (Table 2) and the time events list shown in Fig. 5. These parameters are one embodiment of the N_2_ diversion method described here, and the steps and description will allow The Analyst to successfully reproduce the method but provide all the information required to understand how to modify the method timings, should they require and seek to do so. In this method, the GC column used was 1.5 m long with a 4 mm inner diameter packed with 45-60 mesh 5 Å molecular sieve.

**Table 1:** Recommended Flash/EA IsoLink Method Settings.

Parameter	Parameter Value
Left Furnace	1400 ^o^C
Right Furnace	N/A
Helium Carrier Flow Rate	40 ml/min
Helium Reference Flow Rate	100 ml/min
GC Oven temperature	50 ^o^C
Sampling Delay Time	0 S
Cycle (Run Time)	∼700 s
TCD Filament	On
TCD Polarity	Positive
TCD Baseline	Auto-set to 1000 µv

**Table 2:** Recommended TC/EA Settings.

Parameter	Parameter Value
Jumo Controller (Furnace)	1400 ^o^C
Jumo Controller (GC Column)	50 ^o^C
Helium Carrier Pressure Regulator	0.4 kPa (40 ml/min)
Helium Reference Pressure Regulator	1.1 kPa (100 mls/min)

*ANALYST TIP:* The Analyst can apply knowledge of GC Temperature, carrier gas flow rate and event timing to optimize CO peak shape and/or reduce total analysis time [1], which will provide a range of additional method embodiments relative to the specific GC column length, inner diameter and packing material, and relative to the specified time events list shown in Fig. 5. All readers should note that using a different GC column comparable to the one described here, is likely to produce different chromatography results, including insufficient N_2_ and CO analyte baseline resolution. Using a different GC column may require more trial-and-error tests to successfully implement this N_2_ diversion method, for which the reader can refer to Chen et al. [1] for an overview of chromtography effects. Generally, the ``standard'' GC columns in manufacturer provided EAs require to be changed for an extended length GC column to sufficiently separate N_2_ and CO and thus successfully implement an N_2_ diversion method.

*STEP 1*: In the Acquisition or Workspace module of Isodat, select ``New'' and select ``Method''. When the Method window appears, ensure that the Gas Configuration selected is ``CO'' and under Auto Dilution, select ``Fixed'' and set a value for ``Reference Intensity [mV]'' and ``Sample Dilution [%]''. The sample peak intensity will vary depending upon the elemental concentration of oxygen in the sample, sample weight injected into the reactor, sample dilution applied and instrument sensitivity, amongst other factors.

*ANALYST TIP:* To avoid ion source linearity effects on the raw data, The author recommends matching the intensity of the reference peak and sample peak as closely as possible, and ideally within 500 mV of one another. An operating signal intensity range of 5–8 volts for measured δ^18^O values on CO analyte gas provides repeatable and reproducible data and is one suggested range for The Analyst to work within. Regardless of the working range chosen, The Analyst must ensure the stability of their Mass Spectrometer for their chosen signal intensity range and ensure that the monitoring gas peaks, and sample gas peaks closely match in intensity.

*STEP 2:* Create the time events list in accordance with Fig. 5 as a starting point. This assumes that The Analyst is using the same GC column as started in this method. If the GC column is different, The Analyst can still implement this method, however, it is advised to check previous sample chromatograms, or analyse a sample without a diversion, to determine where the N_2_ and CO baseline resolve and determine the specific timing required for the quantitative N_2_ diversion.
*STEP 3:* In the other tabs of the Method, The Analyst can use with the default values in the ``Compound Names'', ``Evaluation@CO'', ``Peak Detection@CO'' and ``Printout@CO'' tabs. However, if The Analyst uses any specific values in their laboratory in these tabs, they can be implemented here. Once the Method has been completed, the Analyst must save the Method File with an appropriate name, for example ``N2_Diversion_O_Isotopes.met'', or another name as they see fit. Once saved, this Method File can be used in the sample sequence.

*ANALYST TIP:* For the elemental analyzer GC Oven Temperature, the author notes that the low GC column temperature used may result in a more frequent need to bake the GC column between analytical runs. How often a GC baking routine is required depends upon the samples being analysed and their by-products, the GC column length, inner diameter and packing material. It is recommended therefore that The Analyst notes the CO peak retention time and CO peak width/peak intensity, which is taken to mean the time recorded at the CO peak maximum intensity and monitor these during analysis and data evaluation. If peak width becomes higher, peak intensity lower and retention time later, there may be a need to bake the GC column. However, there may also be other maintenance issues that require attention, such as in the reactor or capillary lines between the reactor and GC Column, for example, which may be caused by blockages in the analytical pathway.

*STEP 4:* The Analyst will need to create an EA method file according to Table 1 (if they have a Flash 2000 or EA IsoLink), which must be done in the Isodat Acquisition Module. If The Analyst has a TC/EA, this is manually controlled, and the settings must be used for the JUMO controllers, and the pressure regulators, as stated in Table 2.

*ANALYST TIP:* Every GC based system, in relation to reactors, capillaries and water traps, is unique. After setting the method parameters, The Analyst can check the flow rates exiting the EA using a flow meter: the author recommends attaching a flow meter to the exit capillary that is connected to the ConFlo IV Device, and checking it is the value expected. Prior to doing this, The Analyst is strongly recommended to place the ConFlo IV Device into Standby mode: failure to do this may introduce air from the atmosphere, which will add water and oxygen to the source and may negatively impact filament performance and lifetime, as well as create (at least) temporary background issues.

*STEP 5:* Once the Method file and EA methods are set, The Analyst is required to create a sequence file. In the Acquisition or Workspace module of Isodat, select ``New'' and select ``Sequence''. A dialogue box will appear with a drop-down list for ``Number of Samples'' – The Analyst should determine how many samples are to be analysed and select this number. The sequence file allows The Analyst to enter the sample type, amount, other sundry meta data as required, and select the EA method and Time Event Method. The sequence file can be saved in accordance with local laboratory protocols.

In this proposed method, The Author has opted to add in a monitoring gas peak between the return of the carrier flow from the EA to the mass spectrometer and the evolution of the CO peak from the GC column. This allows an estimation of CO baseline stability, which is briefly summarised in the Method Validation section.

## PART 3: What is happening in the ConFlo Device during the method?

**Fig. 6:**
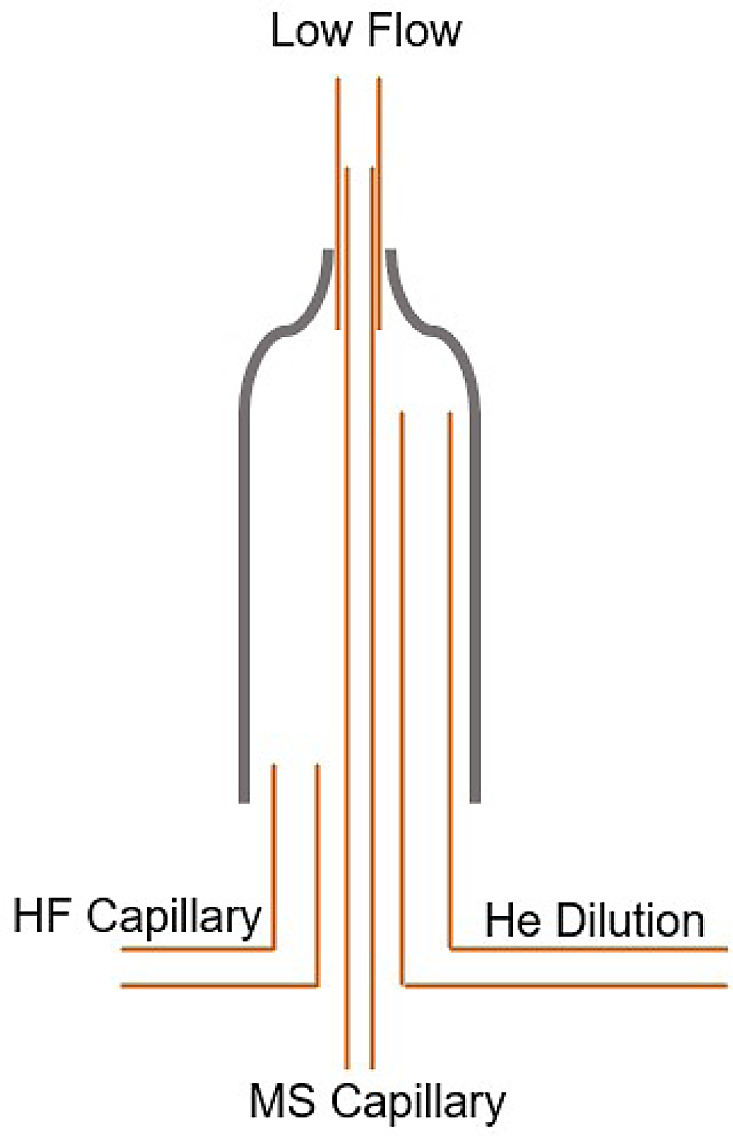
This is the N_2_ Diversion mode where the Sample Open Split at the beginning of the time events list (2–305 s as per Fig. 5) ensures that all gas from the Elemental Analyser is flowing to waste in the ConFlo IV Device and the mass spectrometer capillary is connected to pure helium gas. The configuration remains like this until the N_2_ peak has exited the Elemental Analyser and been vented via the ConFlo IV.

In the context of the time events list (Fig. 5), at the beginning, the LF-On and MS Capillary-On with a green dot at 2 s, and the HF Capillary-On and HF II-On with red dots at 8 s configures the Sample Open Split in the ConFlo IV as shown in Fig. 6. These commands divert the gas flow coming from the EA to vent via the gas extraction system connected to the rear of the ConFlo IV Device. Further, it inserts the mass spectrometer capillary into the low flow capillary at the top of the open split and maintains a helium flow into the ion source. The Analyst should note that this method assumes that there are no other peripheral devices connected to the Low Flow port of the ConFlo IV, and that it is configured in a helium gas loop (however, it coudl reaosnably be assumed that helium gas coming from any other peripheral shoudl not create analytical issues). The author notes that Ext Trig 1 in this method is used to trigger the Costech Zero Blank Autosampler to drop a single sample via the ConFlo IV External Trigger 1 communication port. During this time (0–305 s), the CO analyte is being resolved from the N_2_ analyte by the GC column. The Analyst should note that this approach ensures that the CO analyte remains on the exact background/baseline that it was thermally decomposed on in the reactor.

**Fig. 7:**
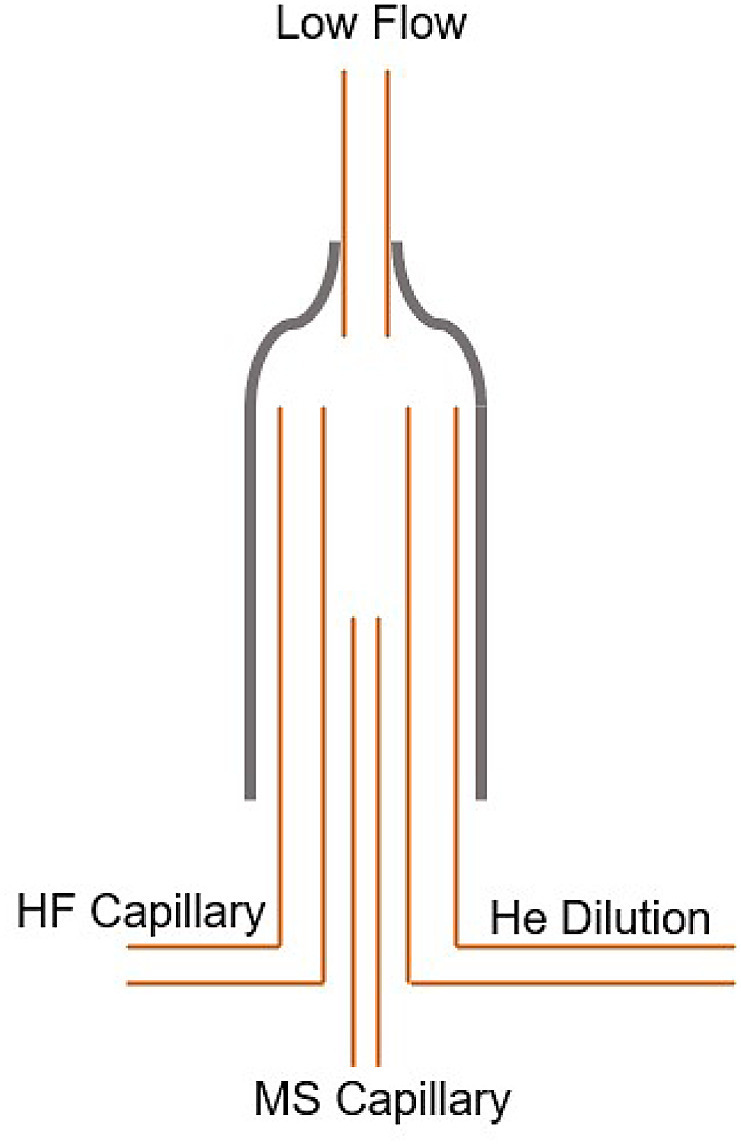
Configuration of the Sample Open Split at 305–306 s as per Fig. 5. At this time, all gas from the Elemental Analyser is flowing to the mass spectrometer and may, or may not, be diluted further by the addition of helium.

From Fig. 5, after 305 s, the HF Capillary and MS Capillary change position, as shown in Fig. 7, which allows the CO analyte gas to be conveyed to the mass spectrometer after it has completely eluted from the GC column.

**Fig. 8:**
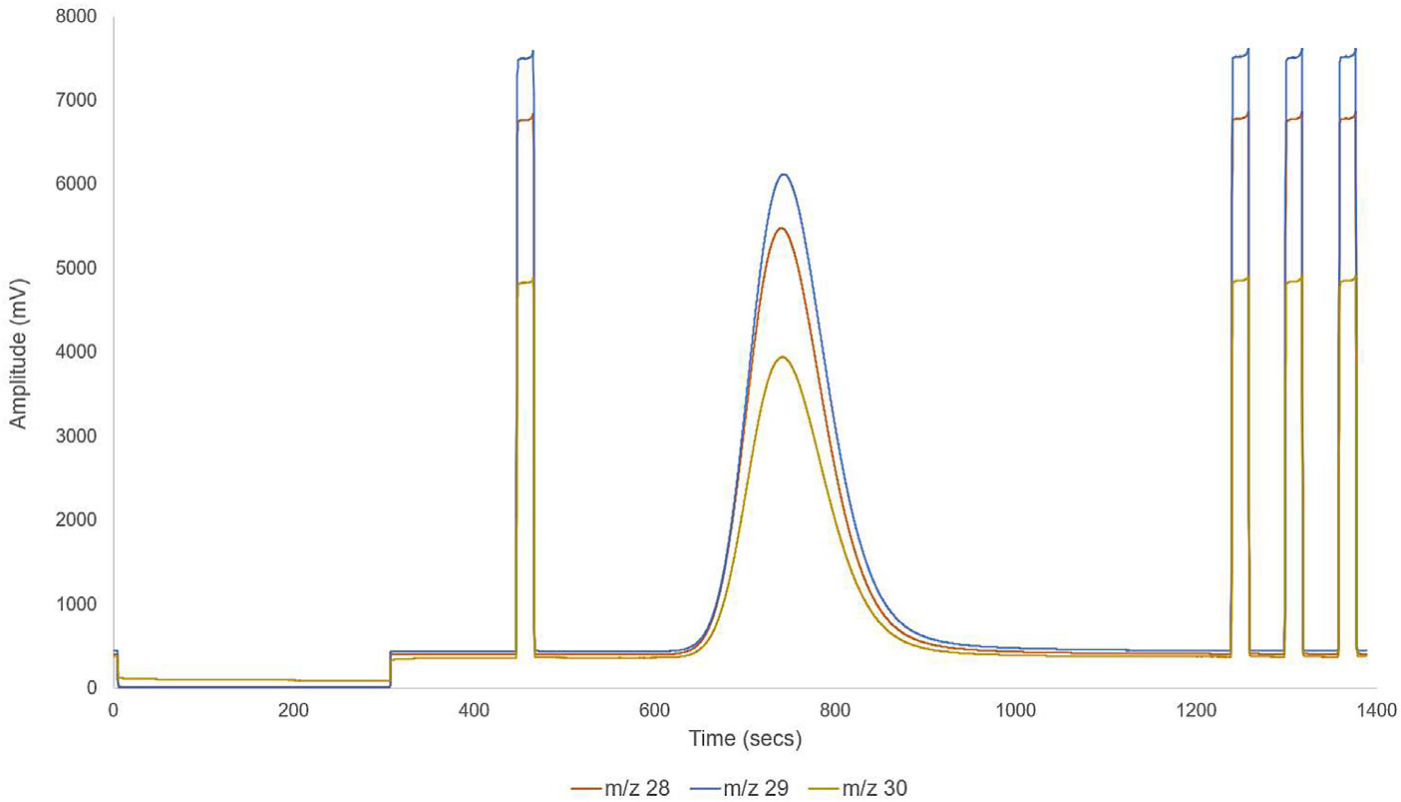
Example chromatogram for the N_2_ diversion method described herein.

The N_2_ diversion method embodiment described herein will produce a chromatogram as shown in Fig. 8.

## PART 4: Method validation

### Baseline stability check

The first step to determine the robustness of the method was to assess the baseline stability after the gas flow from the EA was removed from the ion source (as shown in Fig. 8). This was achieved using a monitoring gas peak before the CO sample gas peak (440–460 s in Fig. 8) and the δ^18^O value calculated relative to the final monitoring gas peak (e.g., 1360–1380 s in Fig. 8). The δ^18^O_VSMOW-SLAP_ value for the monitoring gas was assigned as *-6.32*‰ normalized versus USGS 47.

For the baseline stability monitoring gas peak, the repeatable mean ± 1 standard deviation was -*6.32 ± 0.01‰* for *n* = 9 sample measurements. These data indicate that baseline stability in the mass spectrometer is achieved (i.e., stable) in this method before the CO sample peak elutes from the GC column.

*ANALYST TIP*: The author notes that the baseline stability is good when testing this method, however each system is different on any given day (for multiple reasons). After initial method monitoring by The Analyst, consideration can be given to removing the monitoring gas peak before the CO analyte sample peak, plus one of the monitoring gas peaks at the end of the method. This will allow The Analyst to reduce total analysis time, and close in on the timing achieved by Farqhaur et al. [2]. This is a discretionary option for each Analyst and based on measurements made in their laboratory that give confidence in the stability of the baseline (reproducibility and repeatability).

### Sample data

**Fig. 9:**
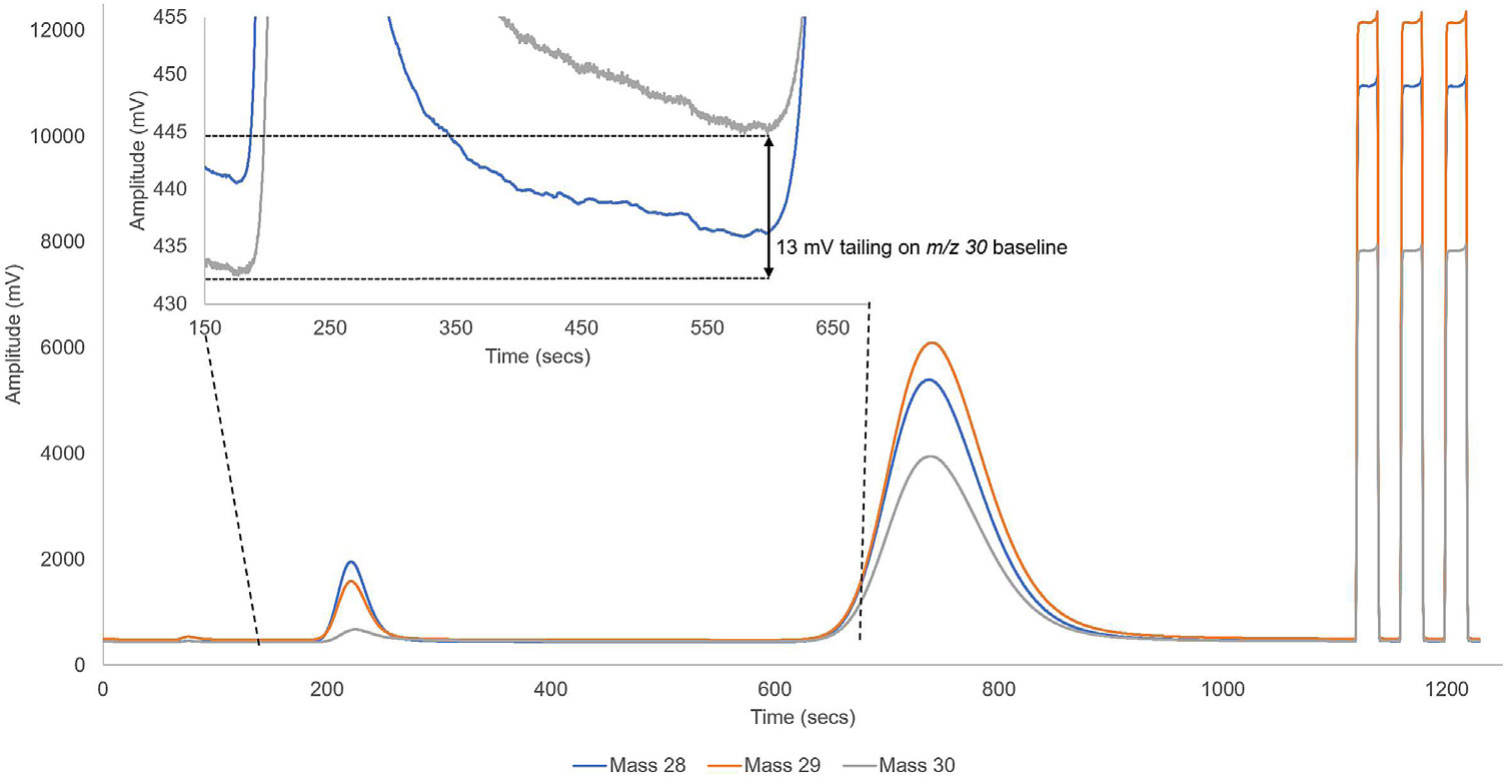
Example chromatogram when N_2_ is not diverted and allowed to tail into the CO sample peak at *m/z* 30.

A test sample material of Gelatin (C_102_H_151_N_31_O_39_), which has approx. 16.8% Nitrogen and approx. 24.1% Oxygen, was analysed. The repeatable mean ± 1 standard deviation for measured δ^18^O_VSMOW-SLAP_ values without diverting N_2_ was *9.21 ± 0.25*‰ (*n* = 6), and after diverting N_2_, was *12.82 ± 0.21*‰ (*n* = 6). Measured δ^18^O_VSMOW-SLAP_ values are reported relative to USGS47 and U04. The *3.61*‰ difference between the repeatable mean values is directly attributable to a 13 mV tailing on the *m/z* 30 of the CO analyte by the N_2_ analyte, which is *0.27‰/mV* for this specific sample type (see Fig. 9). The Analyst should note that the isotopic composition of the nitrogen and oxygen varies between sample materials, as does the total elemental concentration and thus sample weight analysed. These factors all contribute to potential interference on the minor ion trace, and thus the values reported for the Gelatin sample here are not likely to be transferable to other sample matrices or situations. The reader is directed to Additional Information for a short overview of the *m/z* 30 isobaric interference, and for further references on the topic.

### Advantages of this method

Comparable with other known solutions (see Additional Information), the solution described here can be implemented:

i) Without hardware modification (or minimal, if the Analyst decides to change the GC column to a longer one) of the analytical flow pathway, reducing complexity, additional risk of leaks and the potential for memory effects resulting for surface interactions;
ii) Using only the ConFlo IV Universal Interface Device and Isodat Software Suite 3.0;
iii) With no need for software programming, ensuring ease of access for all users.

The author notes that with changes in carrier gas flow rate, GC column temperature, length, inner diameter and packing material and fewer monitoring gas peaks, The Analyst can seek to reduce the total analysis time.

## Additional information

In thermolysis Elemental Analysis Isotope Ratio Mass Spectrometry (EA-IRMS), there is a well-known analytical artefact produced when analysing nitrogen-containing sample materials. This artefact results in highly reproducible, but biased, measured δ^18^O values. The artefact generally arises in Elemental Analysers (EA) that use a static temperature gas chromatography (GC) column: when a nitrogen-rich sample material (containing >0.5% w/w nitrogen alognside the oxygen content) is introduced into the reactor, two of the analytes that are conveyed out of the reactor and onto the GC column are Nitrogen (N_2_) and Carbon Monoxide (CO). In EA systems that are not optimised for such nitrogen-rich sample types (which, as standard, they tend not to be), the static temperature GC column generally does not provide baseline resolution of the N_2_ and CO analytes, meaning that the measured δ^18^O values on the CO analyte are affected by the interference of the N_2_ analyte tailing onto the CO analyte peak. This lack of baseline resolution results in an isobaric interference in the mass spectrometer.

When N_2_ analyte gas is permitted to enter the ionization chamber of the ion source in the mass spectrometer, with insufficient resolution with the CO analyte, it generates an isobaric interference at *m/z* 30. The N_2_ and CO analytes have a *m/z* 28, *m/z* 29, and *m/*z 30, which the mass spectrometers commonly used in EA-IRMS cannot resolve: The primary analyte of interest, CO, as [^12^C^16^O]+ (*m/z* 28), [^12^C^17^O]+ and [^13^C^16^O]+ (*m/z* 29) and [^12^C^18^O]+ (*m/z* 30) and N_2_, as [^14^N^14^N]+ (*m/z* 28) and [^14^N^15^N]+ (*m/z* 29), and [^14^N^16^O]+ (*m/z* 30). Measured δ^18^O values of the sample material are calculated from counting [^12^C^16^O]+ (*m/z* 28) and [^12^C^18^O]+ (*m/z* 30): the minor ion current, and thus limiting factor in precision, being on *m/z* 30 for the CO analyte. In the case of N_2_, the isobaric interference at *m/z* 30 is (almost entirely) present in the ion source as nitrous oxide as [^14^N^16^O]+. The amount of [^14^N^16^O]+ produced is a function of the amount of N_2_ entering the ion source, and the amount of O_2_ and H_2_O in proximity with the filament: it is highly variable, instrument to instrument, and within instruments on different days. Unlike [^14^N^14^N]+ and [^14^N^15^N]+, [^14^N^16^O]+ behaves differently and is relatively slower to be removed from the analytical stream, resulting in a strong tailing effect on *m/z* 30.

The *m/z* 30 deriving from [^14^N^16^O]+ causes incorrect measured δ^18^O values to be produced because the *m/z* 30 ion current appears to detect more ions, which the instrument software incorrectly assumes is entirely from [^12^C^18^O]+: this is exacerbated by the fact the [^14^N^16^O]+ has a substantially different isotope composition, for example, *-680*‰, meaning a small influence on *m/z* 30 can cause a significant change in the measured δ^18^O values (i.e., a bias consequent of an analytical artefact). In this study, the mass spectrometer used has a mass resolution ≤110, meaning that the [^12^C^18^O]+ and [^14^N^16^O]+ are not differentiated from one another by the mass analyser. To fully resolve this isobaric interference, a mass spectrometer would require a theoretical resolving power of at least 25600 to resolve the coincident ions [^12^C^18^O]+ and [^14^N^16^O]+ at *m/z* 30, which is considerably greater than the Isotope Ratio Mass Spectrometer used in this study (and other similarly manufactured mass spectrometers traditionally used for EA-IRMS).

Therefore, avoiding the [^14^N^16^O]+ isobaric interference on the CO Analyte is not only highly desired, but also critical, especially when there is an option for The Analyst to keep the sample evolved CO analyte on the background unto which it evolved. This, together with avoiding N_2_ entering the ion source should be the aim of every Analyst seeking to measure δ^18^O values on nitrogen-rich sample materials. It is readily achievable and shown in this method to be cost-free and routine. There should *not* be a reliance upon matrix matching of sample materials and quality control materials to ``deal with the effect'' via correction, or any other such post-measurement data corrections/calculations on data that are known to be reproducible wrong. Such appraoches only serve to introduce greater uncerintaty on the data, which ultimately, can be avoided.

### Brief overview of documented solutions for N_2_ and CO baseline separation

To resolve the baseline resolution challenge in EA's that use static temperature GC columns, several solutions have been proposed including (i) the diversion of the N_2_ analyte to waste such that no N_2_ enters the mass spectrometer [3,4,5], (ii) applying the maximum dilution of an interface device to minimise, but not eliminate, the N_2_ entering the mass spectrometer [6], (iii) use of a longer GC column to achieve baseline resolution of N_2_ and CO analyte peaks [7], and (iv) manual correction of raw peak area data [6,7] to remove the minor ion current interference on the m/z 30 peak area of the measured CO analyte trace. Each solution has merit when directly compared to methods that measure δ^18^O values with an N_2_ interference on the *m/z* 30 ion current.

The author notes the existence of purge and trap technology from Elementar that has proposed an N_2_ diversion solution through CO adsorption and thermal desorption in a time event manner [8]: however, this has not been explored here. Additionally, but separately, the method described herein does not permit the sequential measurement of δ^2^H values with δ^18^O values of nitrogen-containing samples: the latter point has additional considerations as noted by Meier-Augenstein et al. [9] and Nari et al [10], including overviews of the Cr reactor technique.

### What does the chromatogram look like without a N_2_ diversion?

For visual comparison with the N_2_ diversion shown in Figs. 8, 9 demonstrates the *m/z* 30 tailing of the N_2_ peak into the CO. The chromatogram is representative of the data collected in this study when N_2_ was not diverted, giving a 13 mV tailing effect on the CO *m/z* 30 trace.

## Ethics statements

No relevant statements.

## CRediT authorship contribution statement

**Christopher Brodie:** Conceptualization, Methodology, Investigation, Validation, Writing – original draft, Writing – review & editing, Project administration, Formal analysis.

## Declaration of Competing Interest

The author declares that they have no known competing financial interests or personal relationships that could have appeared to influence the work reported in this paper.

## Data Availability

This dataset is very small used only to demonstrate the point. Other publications, listed in the references, have shown this already, nullifying the need for a substantial dataset.
